# Rheological and Microstructural Evaluation of Collagen-Based Scaffolds Crosslinked with Fructose

**DOI:** 10.3390/polym13040632

**Published:** 2021-02-20

**Authors:** Pablo Sánchez-Cid, Mercedes Jiménez‑Rosado, Victor Perez-Puyana, Antonio Guerrero, Alberto Romero

**Affiliations:** 1 Department of Chemical Engineering, Facultad de Química, 41012 Sevilla, Spain; pabsanbue@alum.us.es (P.S.-C.); alromero@us.es (A.R.); 2 Department of Chemical Engineering, Escuela Politécnica Superior, 41011 Sevilla, Spain; mjimenez42@us.es (M.J.-R.); aguerrero@us.es (A.G.)

**Keywords:** aerogel, collagen, fructose, hydrogel, scaffold, tissue engineering

## Abstract

In recent years, tissue engineering research has led to the development of this field by designing scaffolds with better properties that can fulfill its purpose of better and faster tissue regeneration, consequently improving people’s quality of life. Scaffolds are matrices, predominantly composed of polymeric materials, whose main function is to offer support for cell adhesion and subsequent growth, leading to the regeneration of the damaged tissue. The widely used biopolymer in tissue engineering is collagen, which is the most abundant protein in animals. Its use is due to its structure, biocompatibility, ease of modification, and processability. In this work, collagen-based scaffolds were developed with different concentrations and processing techniques, by obtaining hydrogels and aerogels that were characterized with an emphasis on their morphology and mechanical properties. Moreover, fructose was added in some cases as a chemical crosslinking agent to study its influence on the scaffolds’ properties. The obtained results revealed that the scaffolds with higher collagen concentrations were more rigid and deformable. Comparing both systems, the aerogels were more rigid, although the hydrogels were more deformable and had higher pore size homogeneity. Fructose addition produced a slight increase in the critical strain, together with an increase in the elastic modulus.

## 1. Introduction

Tissue engineering (TE) was defined as “a multidisciplinary field which applies principles and methods of engineering and life sciences for the development of biological substitutes, to restore, maintain or improve tissue function” by Martin et al., (2004) [1]. TE is based on the development of 3D structures with a high porosity (scaffolds) to provide an ideal environment for the regeneration of tissues and organs. These scaffolds are biocompatible matrices fundamentally designed to provide the biological needs of the cells involved, as well as to offer a template for new tissue formation. Furthermore, they also contribute to the mechanical and structural integrity of the treated region [2,3,4].

In this study, two types of scaffolds were synthesized and characterized, namely hydrogels and aerogels. According to Hoffman (2012), hydrogels are “hydrophilic polymer networks that can absorb between 10–20% and up to thousands of times their dry weight in water. Hydrogels may be chemically stable, or they may degrade and eventually disintegrate and dissolve.” They are called ‘chemical’ or ‘physical’ depending on their intermolecular interactions [5]. Aerogels are solid systems with meso-and-macropores, whose diameter can be up to a few hundred nanometers. In addition, aerogels have a high porosity (up to 95%) being their dispersed phase a gas. Some studies point out that it is possible to manufacture aerogels at room temperature drying conditions [6], although supercritical conditions are required in most cases [7].

TE involves the design and modification of biomaterials that prompt the formation of tissue in a biomimetic environment with appropriate biomechanical properties [8]. Polymers are the most promising materials to obtain these requirements, because they are available in a wide variety of compositions, properties, and shapes, and allow the processing of complex structures. In this way, the use of natural polymers, such as proteins and polysaccharides, is especially interesting, since they are main components of or have similar macromolecular properties to the native extracellular matrix (ECM) [9,10], e.g., proteins such as collagen [11], elastin [12] or even combinations of both proteins [13]. Collagen is the biopolymer that is the most abundant in animals and, therefore, it is one of the most used materials in TE for scaffold production.

Among the different types of collagen, fibrillar collagen is the most abundant in vertebrates, playing a structural role. This role consists of giving molecular architecture, shape, and resistance to tissues, like tensile strength in the skin or resistance to traction in ligaments [14]. Its suitability in TE applications is based on being biocompatible, bioresorbable, and non-immunogenic. It can be processed in different forms (sheets, sponges, foams, powders, nanofibrous matrices, etc.) and it is soluble in acidic solutions. Furthermore, a key factor in TE as the degradation rate can be modulated using different strengthening methods like enzymatic pre-treatment or crosslinking modifications [15,16].

Crosslinking is the formation of new bonds or relatively short sequences of chemical bonds to link polymer chains [17]. Crosslinking may occur during the polymerization reaction, using the appropriate monomers, or after the polymerization step by using crosslinking agents that stimulate the necessary reactions to bind different polymer molecules. The importance of crosslinking procedures lies in the improvement of high-temperature stability and mechanical properties, leading to optimal materials for industrial use [18]. The resulting modifications in the structure strongly depend on the crosslink-density [17]. 

Natural compounds that could act as chemical crosslinking agents are of special interest since they do not decrease the biocompatibility of the scaffold, e.g., fructose, which is a carbohydrate present in different vegetables, fruits, and honey. It is a monosaccharide with the same structure as glucose since they are isomers [19]. Fructose is obtained by using microbial enzymes to hydrolyze the starch extracted from different cereals and transform it into glucose, which, through an isomerization process, is converted to fructose, specifically d-fructose [20]. Fructose, and generally sugars, can undergo a chemical crosslinking by the so-called non-enzymatic glycation or Maillard reaction [21,22]. Maillard reaction is a chemical and non-enzymatic reaction that is produced in three main steps [23]. First, a condensation reaction between the carbonyl group of fructose and the amino group of proteins takes place, generating a Schiff base. Due to the instability of the Schiff base, it is subsequently transformed into the protein Amadori product [24]. Finally, the Amadori compound drives to the formation of advanced glycation end-products, commonly known as AGE products [25].

The principal objective of this work was to develop collagen-based scaffolds with suitable morphological and mechanical properties for their potential application in muscular tissue growth. To achieve the global purpose, some specific objectives were proposed: (1) the development of hydrogels and aerogels with the same processing protocol; (2) comparative evaluation of the mechanical and morphological properties for both hydrogels and aerogels systems with different collagen concentrations; and (3) the evaluation of the incorporation of fructose as a natural crosslinking agent to modified the mechanical and morphological properties of the chosen collagen aerogel.

## 2. Materials and Methods 

### 2.1. Materials

Porcine collagen (Type I, protein content higher than 95 wt.%) was purchased from Essentia Protein Solutions (Graasten, Denmark). Acetic acid and fructose (≥99%) were both supplied by Sigma-Aldrich (Steinheim, Germany).

### 2.2. Formation of Scaffolds

The scaffolds were obtained following the procedure described by Perez-Puyana et al. [26]. The same preparation procedure was carried out for both systems (hydrogels and aerogels), with the difference that, to obtain aerogels, two further steps were necessary for water removal after hydrogel formation (freeze-drying process). A gelation process was conducted to obtain the hydrogels. The process followed was similar to that used in other studies on protein-based hydrogels [26]. Briefly, collagen solutions were prepared at different concentrations (namely 5, 10, and 20 mg/mL) using acetic acid as solvent (0.05 M, pH = 3). Subsequently, the solutions were centrifuged at 12,000 rpm and 4 °C (MEDIFRIGER BL-S, J.P Selecta, Barcelona, Spain).

Once centrifuged, the liquid phase was separated and kept in a fridge (Equitec, Madrid, Spain) at 4 °C for 1 h. After this, the formation of the hydrogels was completed. The aerogels were later obtained by freeze-drying (<15 Pa for 24 h), with a solvent removal by sublimation, using a freeze dryer (LyoQuest, TELSTAR, Barcelona, Spain).

For the crosslinking study with fructose, the 10 mg/mL collagen aerogel was selected, and different fructose concentrations (10 and 40 wt.% from the respective collagen concentration) were added to the solutions at the first step of the procedure (hydrogels formation). This was the same as the one for the scaffolds without a crosslinking agent. Finally, their structural and mechanical properties were studied and compared.

### 2.3. Scaffold Characterization

#### 2.3.1. Hydrogel Characterization

##### Rheological Evaluation

In hydrogels, the viscoelastic properties were evaluated using two types of rheological tests previously described in Perez-Puyana et al. (2020) [26]. Briefly, strain sweep tests (0.1–100% at 1 Hz and 20 °C) were carried out to determine both the linear viscoelastic range (LVR) and critical strain. Moreover, frequency sweep tests (0.02–20 Hz at 20 °C and a specific strain from the LVR) were also performed. In these tests, G′, G″ and η* values (elastic and viscous moduli and complex viscosity, respectively) were measured and compared. This characterization was carried out in a AR 2000 oscillatory rheometer (TA Instruments, New Castle, DE, USA) with serrated parallel plate geometry (diameter: 40 mm).

##### Morphological Evaluation

The hydrogels were morphologically assessed using a Cryo-SEM microscope (Zeiss-EVO, Oberkochen, Germany), at an acceleration voltage of 10 kV. All samples were firstly cooled with nitrogen at −196 °C and sputtered with Au. Image-J software (National-Institute for Health, Bethesda, MA, USA), was used to evaluate both mean pore size and its distribution for the selected systems.

#### 2.3.2. Aerogel Characterization

##### Rheological Evaluation

Dynamic compression tests were carried out following the procedures described by Perez-Puyana et al., (2019) [27]. An RSA3 rheometer (TA Instruments, New Castle, DE, USA), with a smooth parallel-plate geometry (dia: 15 mm) was used to perform the strain sweep tests (2.5 × 10^−4^–2.5% at 1 Hz and 25 °C) and the frequency sweep tests (0.02–20 Hz at 25 °C and a specific strain from the LVR). In these tests, E′ and E″ (elastic and viscous moduli) together with tan δ (loss tangent) and μ* (complex viscosity) values were measured.

##### Morphological Evaluation

The aerogels were morphologically analyzed using a Zeiss EVO-SEM microscope (Zeiss-EVO, Oberkochen, Germany) at an acceleration voltage of 20 kV. All samples were previously treated with Osmium vapor (1%) in a fumehood for 8 h to fix the samples and facilitate their observation under the microscope. Later, the fixed samples were covered by a thin film of Au, using a sputter coater Leica (Wetzlar, Germany), to improve the quality of the micrograph (improving the sample conductivity). The images were analyzed (mean pore size and pore size distribution) using a free domain software (Image-J, National-Institute for Health, Bethesda, MA, USA).

##### Crosslinking Degree

Crosslinking degree was quantified following the protocol of Ofner and Bubnis [28]. Briefly, it was obtained by measuring the content of free and crosslinked amino groups in the different systems (Genesys-20 Thermo Scientific, Waltham, MA, USA). The quantification was obtained using a blank as 100% of crosslinked amino groups, while a system without adding fructose was used as 0% of crosslinking induced. In this way, the crosslinking degree induced by the addition of fructose was evaluated.

##### Swelling Degree

This property is important to predict the behavior of the scaffold in the body, where it will be in close contact with biological fluids. For their evaluation, the scaffolds were immersed in distilled water at 25 ± 2 °C, weighing them after 24 h [29]. The swelling ratio was calculated using Equation (1):

(1) $$\mathrm{Swelling}\;\mathrm{ratio}\;\left(\%\right)=\left(\frac{w-w_{0}}{w_{0}}\right)\times 100$$

where $w_{0}$ and w are the weights of the scaffold before and after the water immersion, respectively.

### 2.4. Statistical Analysis

Each measurement was performed in triplicate. Statistical analyses were performed using PASW-Statistics (Windows, Version18: SPSS, Chicago, IL, USA). *t*-tests and one-way analysis of variance (*p* < 0.05) were used for each analysis. Standard deviations were obtained for the parameters evaluated.

## 3. Results and Discussion

### 3.1. Hydrogels vs. Aerogels at Different Collagen Concentrations

**Figure 1:**
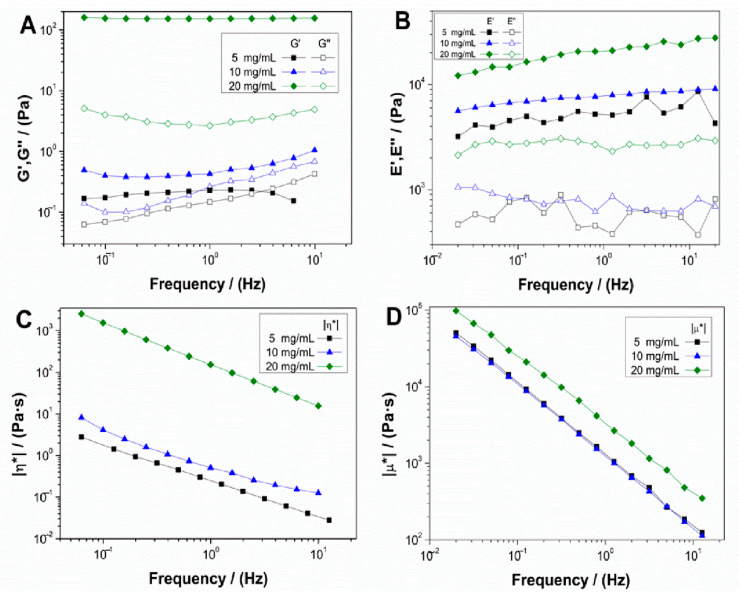
Evolution of elastic (G′ and E′) and viscous (G″ and E″) moduli and complex viscosity (η* and μ*) of collagen-based hydrogels (**A**,**C**) and aerogels (**B**,**D**) at different collagen concentrations (5, 10 and 20 mg/mL).

Figure 1 shows the evolution of the elastic and viscous moduli (Figure 1A,B, respectively) and complex viscosity (Figure 1C,D) with frequencies for both hydrogel (Figure 1A,C) and aerogel (Figure 1B,D) systems, with different concentrations of collagen (5, 10 and 20 mg/mL).

Both systems showed a gel-like behavior, where the elastic modulus was above the viscous modulus, without great variations in frequency. These results indicated that all the systems had enough stability, allowing prediction of their behavior regardless of the time of strain application. Besides, although no significant differences were observed in the spectrum tendencies with frequency, an increase in collagen concentration, especially for 20 mg/mL, resulted in an increment of the corresponding elastic and viscous moduli. This behavior with the increase of biopolymer concentration has been previously observed in other works [30]. For complex viscosity (Figure 1C,D), the same tendency can be observed, where concentrations of 5 and 10 mg/mL almost overlap, although the 20 mg/mL scaffolds show a significant increase for both hydrogel and aerogel systems, which means that higher concentrations result in a more viscous behavior.

Table 1 shows the mean values of critical strain (γ_crit_), G′, tanδ, and η* at 1 Hz for hydrogel systems and critical strain (γ_crit_), E′, tanδ, and μ* at 1 Hz for aerogel systems. From these results, it can be confirmed that, with the increase of collagen concentration, there were no significant differences between the 5 and 10 mg/mL systems. However, the 20 mg/mL scaffolds had higher moduli, viscosity, and critical strain, indicating more rigid, viscous, and deformable hydrogel and aerogel at higher collagen concentration. In addition, it is remarkable that the aerogel systems were more rigid and viscous than the hydrogel systems with the same concentrations. Nevertheless, the hydrogel system with the highest concentration was more deformable than the aerogel system with the same concentration.

**Figure 2:**
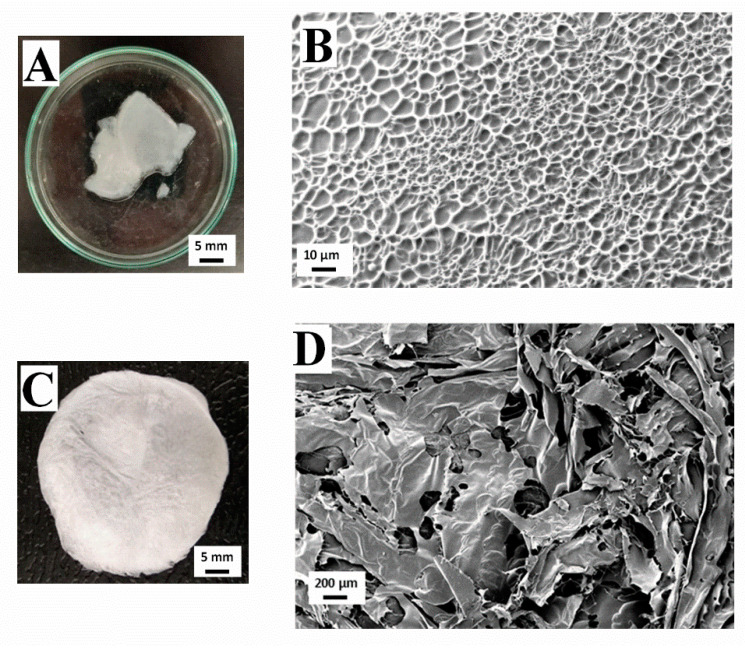
Macroscopic and microscopic images of a collagen-based hydrogel (**A**,**B**) and a collagen-based aerogel (**C**,**D**) at a concentration of 10 mg/mL.

Figure 2 shows a macroscopic and microscopic view of collagen-based hydrogel and aerogel, both at a concentration of 10 mg/mL. Figure 2A shows the macroscopic view of the 10 mg/mL collagen hydrogel, which can be described as a white gel texture structure. The microstructure analyzed by Cryo-SEM of the same hydrogel is represented in Figure 2B. As can be observed, there is an important degree of structuration with a much more homogeneous pore size distribution compared to the aerogel with the same concentration (Figure 2D), as the scaffold presents higher porosity with a large amount of great-sized pores, since its dispersed phase is a gas, whereas, for the hydrogel, the dispersed phase is liquid. This would explain the difference of structures between both systems. However, the main distinctive feature in the case of aerogels is the heterogeneity in the size of the pores, which are comprised in the range of 130–300 μm, to be expected for collagen aerogels (120–200 μm) [31]. Furthermore, it is worth highlighting the high interconnectivity degree between pores, which, along with its propitious pore size, would be favorable for cell implantation and growth, and the consequent tissue regeneration. Moreover, there is greater uniformity in the pore size of the hydrogels (Figure 2B), although it is remarkable that the pores with greater size seem to appear in the outer regions of the hydrogel, whereas the smaller ones are located in inner regions. The pores in the image have an approximate size of 1–11 μm, which is out of the range for favorable muscular tissue cell proliferation (20–125 μm) [32]. For pore sizes under this range, as in this case, cells were excluded from the interior of the matrix, which would pose a problem for its potential use in TE.

Figure 2C shows a picture of the macroscopic appearance of the 10 mg/mL collagen aerogel. It is characterized by an intense white color with certain translucency due to the high porosity of this kind of system. Between Figure 2A,C, there is a marked difference in textures due to the presence (hydrogel) or absence (aerogel) of water in its structure. However, it can be observed that the processing conditions did not affect the coloration of the scaffolds.

### 3.2. Evaluation of Fructose as Crosslinking Agent

After comparing the mechanical and morphological properties of both systems, aerogel was selected for the crosslinking study with fructose, due to its greater rigidity and viscosity, and its better pore size range for muscular tissue cell proliferation. The intermediate concentration was chosen for the evaluation. Fructose has been selected as a chemical crosslinking agent based on the fact that any reducing sugar could induce a Maillard reaction in protein molecules [33,34]. Specifically, fructose would react with the lysine residue in collagen inducing a modification of the microstructure and, therefore, the properties of the aerogels. 

**Figure 3:**
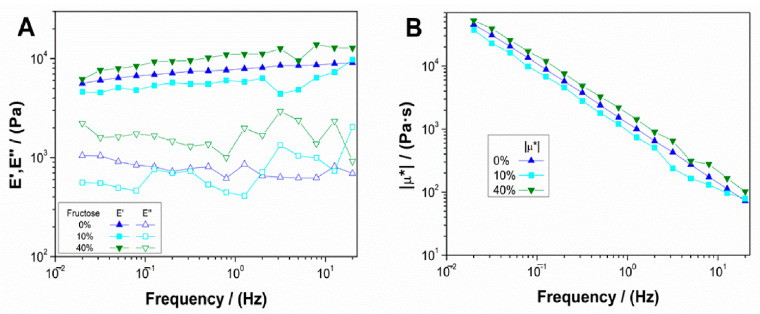
Evolution of (**A**) elastic and viscous moduli and (**B**) complex viscosity for 10 mg/mL collagen-based aerogels with different concentrations of fructose (0, 10 and 40 wt.%).

Figure 3 shows the results of frequency sweep tests for 10 mg/mL collagen aerogels with different fructose concentrations (10 and 40 wt.%), as previously described. The collagen aerogel system without fructose was also included as a reference. Results showed that there was a predominantly elastic behavior with a low influence of frequency (Figure 3A), although certain instability appeared at the scaffold with the lowest fructose concentration. For the system with a lower concentration of added fructose (10 wt.%), the elastic modulus decreased with respect to the aerogel without a crosslinking agent, whereas for higher fructose concentrations there was a slight increase in the elastic modulus. This could be because low crosslinking agent concentrations added to the systems start the crosslinking reaction, which does not provoke the strengthening of the structure. On the other hand, a higher fructose concentration implied a sufficient crosslinking degree that implied a slight improvement of the mechanical properties. Figure 3B represents the results of the variation of the complex viscosity moduli of each system with frequency. As can be observed, the lower fructose concentration aerogel is less viscous (since the elastic modulus value is lower), although similar to the scaffold without a crosslinking agent. However, increasing fructose concentration led to a more viscous behavior, slightly surpassing the values obtained by the collagen aerogel without fructose.

Table 2 shows the values of critical strain (γ_crit_), E′, tan δ and μ* at 1 Hz (E′_1_, tan δ_1_ and μ*_1_) for the aerogel systems with and without fructose. From these results, it can be highlighted that for the low concentration of fructose added, there was a slight increase in critical strain. However, it is worth mentioning that its value was in the error range, implying that there was no significant variation in critical strain for the value obtained for the aerogel without fructose. Moreover, there was a decrease in the elastic modulus and complex viscosity modulus, which meant that the addition of low fructose concentration led to less rigid and viscous scaffolds. Nevertheless, for the 40 wt.% fructose system, there was an increase in elastic modulus and complex viscous modulus, obtaining values closer to those of collagen 20 mg/mL. However, fructose incorporation as a crosslinking agent generates a very slight reaction in the system, thereby adding other chemicals would probably stimulate the reaction [35]. In any case, this increment in rigidity and viscosity does not imply an increase in its critical strain, which establishes that the deformability of the system is not altered along with its biocompatibility, since fructose is a natural product.

**Figure 4:**
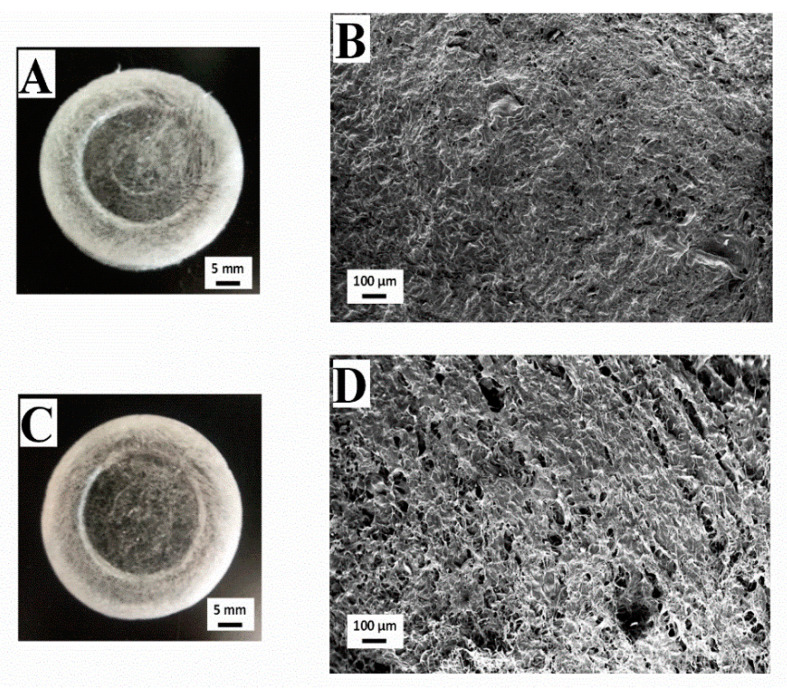
Macrographs and micrographs of 10 mg/mL collagen-based aerogels with different concentrations of fructose: 0 (**A**,**B**), 10 (**A**,**B**) and 40 wt% (**C**,**D**).

Figure 4 shows images of macroscopic and microscopic views of aerogels with different fructose concentrations (10 and 40 wt.%). It is worth highlighting the difference between the macroscopic views of Figure 4A,B, which represent fructose added aerogels, and Figure 2C (system without a cross-linking agent). In this way, the scaffolds with fructose incorporated presented a certain yellowish tonality, which was more noticeable with a higher fructose concentration. This coloration is due to the Schiff base formation after the Maillard reaction with collagen [35]. It can also be noted that this effect involves an increase of the scaffolds’ transparency. Moreover, fructose addition modified the microstructure of the scaffolds, comparing the SEM images obtained (Figure 4B,D) and the reference collagen aerogel without a crosslinking agent (Figure 2D). The scaffold without a crosslinking agent shows a sheet-like microstructure, with great heterogeneity in pore size and distribution, whereas the presence of fructose derived in a more structured and homogeneous microstructure. According to the images included in Figure 4 (Figure 4B,D for the systems with fructose concentrations of 10 and 40 wt.%, respectively), the addition of fructose at 10 wt.% generated a porous structure with pore sizes in the interval of 6–55 µm (mean pore size: ca. 28 µm). Higher fructose concentration (40 wt.%) generated a structure with bigger, circular, and heterogeneous pores (pore size range between 17 and 110 µm and mean pore size of ca. 45 µm), although, as was previously observed, the appearance of this more porous microstructure did not influence the mechanical properties of the system. Overall, SEM imaging revealed that collagen microstructure notably changed by the addition of fructose due to the possible aggregation that occurred due to the Maillard reaction between collagen and fructose. Similar results were found in previous studies carried out by Etxabide et al. [24].

Table 3 shows the values of crosslinking degree and swelling degree of the aerogels with added fructose compared to the reference system without a crosslinking agent. For the crosslinking degree, there was an increasing tendency with fructose concentration, whereas the swelling degree showed the opposite behavior, i.e., when reaching a higher crosslinking degree, the scaffolds were able to absorb less water within their structure, which has been previously observed by Perez-Puyana et al. [36].

## 4. Conclusions

Collagen-based hydrogels and aerogels with adequate mechanical properties for potential use in TE were developed. From the morphology of both systems, different conclusions can be drawn, as the morphology of the aerogel systems was suitable for applications in TE, whereas the hydrogels had problems associated with pore size.

From the results of this study, it can be concluded that higher collagen concentrations lead to better rigidity and deformability. Moreover, the comparison of mechanical properties between the two systems (hydrogels and aerogels) evidenced that the aerogels were more rigid than the hydrogels, whereas the opposite occurred when comparing their deformability. The morphology study proved that both systems had great porosity, although the hydrogels had greater homogeneity in pore size than the aerogels. However, the hydrogels pore size (1–11 μm) did not fit the critical range for muscular tissue cell proliferation (20–125 μm), unlike the aerogels (130–300 μm). Thus, it would be necessary to modify the system composition or processing conditions to overcome this limitation. Fructose addition in the aerogels induced slight changes in both the morphological and mechanical properties of the scaffolds, which seemed to be more evident for higher fructose concentrations (40 wt.%).

Future works will be based on a more detailed study on the application of these scaffolds in tissue engineering. For this, degradation analysis, cell viability, and biological studies in vitro and in vivo will be performed.

## Figures and Tables

**Table 1:** Critical strain, elastic modulus, complex viscosity, and loss tangent at 1 Hz (G′_1_, tan δ_1,_ and η*_1_) of collagen-based hydrogels and aerogels at different collagen concentrations (5, 10, and 20 mg/mL).

Scaffold	Concentration (mg/mL)	γ_crit_ (%)	G’_1_/E’_1_ (Pa)	|η*_1_|/|µ*|_1_ (Pa·s)	tan(δ)_1_
Hydrogel	5	0.6 ^a^	0.13 ^A^	0.13 ^γ^	0.7 ^I^
	10	1.0 ^ab^	0.4 ^A^	0.5 ^δ^	0.6 ^I^
	20	10 ^c^	144 ^B^	147 ^ε^	0.02 ^II^
Aerogel	5	0.8 ^a^	5284 ^C^	1349 ^α^	0.10 ^III^
	10	1.3 ^ab^	6076 ^C^	1270 ^α^	0.09 ^III^
	20	2.0 ^b^	20825 ^D^	3413 ^β^	0.12 ^III^

Different letters (a–b; A–D; α–δ; I–III) as superscripts were included to denote significant differences in the values shown in each column (*p* < 0.05).

**Table 2:** Critical strain (y_crit_), elastic modulus, complex viscosity, and loss tangent at 1 Hz (E′_1_, |µ*|_1_ and tan(δ)_1_, respectively) of 10 mg/mL collagen-based aerogels with different concentration of fructose (0, 10, and 40 wt.%). Different letters as superscripts were included to denote significant differences in the values shown in each column (*p* < 0.05).

Collagen Concentration (mg/mL)	Fructose Concentration (%)	γ_crit_ (%)	E′_1_ (Pa)	|µ*|_1_ (Pa·s)	tan (δ)_1_
5	-	0.8 ^a^	5284 ^A^	1349 ^α^	0.10 ^I,II^
10	0	1.3 ^ab^	6076 ^A^	1270 ^α^	0.09 ^I,II^
	10	2.0 ^ab^	5912 ^A^	969 ^α^	0.07 ^I^
	40	0.8 ^a^	11,567 ^B^	1897 ^β^	0.12 ^II^
20	-	2.0 ^b^	20,825 ^C^	3413 ^γ^	0.12 ^II^

Different letters (a–b; A–C; α–γ; I–II) as superscripts were included to denote significant differences in the values shown in each column (*p* < 0.05).

**Table 3:** Crosslinking and swelling degrees and pore size range for 10 mg/mL collagen-based aerogels with different concentrations of fructose (0, 10, and 40 wt.%). Different letters as superscripts were included to denote significant differences in the values shown in each column (*p* < 0.05).

Scaffolds	Crosslinking Degree (%)	Swelling Degree (%)	Pore Size Range (µm)
Collagen 10 mg/mL (0 wt.% Fructose)	-	113 ^A^	130–300 ^α^
Collagen 10 mg/mL (10 wt.% Fructose)	18 ^a^	70 ^B^	6–55 ^β^
Collagen 10 mg/mL (40 wt.% Fructose)	27 ^b^	32 ^C^	17–110 ^β^

Different letters (a–b; A–C; α–β) as superscripts were included to denote significant differences in the values shown in each column (*p* < 0.05).

## Data Availability

The data presented in this study are available on request from the corresponding author.
